# Pharmacological characterization of GABA_A_ receptors in taurine-fed mice

**DOI:** 10.1186/1423-0127-17-S1-S14

**Published:** 2010-08-24

**Authors:** William J L’Amoreaux, Alexandra Marsillo, Abdeslem El Idrissi

**Affiliations:** 1 Department of Biology, College of Staten Island, 2800 Victory Blvd., Staten Island, NY 10314, USA; 2 Advanced Imaging Facility, College of Staten Island, 2800 Victory Blvd., Staten Island, NY 10314, USA; 3 City University of New York, Doctoral Program in Biology – Neuroscience, 365 Fifth Avenue, New York, NY 10016-4039, USA; 4 Center for Developmental Disabilities, College of Staten Island, 2800 Victory Blvd., Staten Island, NY 10314, USA

## Abstract

**Background:**

Taurine is one of the most abundant free amino acids especially in excitable tissues, with wide physiological actions. Chronic supplementation of taurine in drinking water to mice increases brain excitability mainly through alterations in the inhibitory GABAergic system. These changes include elevated expression level of glutamic acid decarboxylase (GAD) and increased levels of GABA. Additionally we reported that GABA_A_ receptors were down regulated with chronic administration of taurine. Here, we investigated pharmacologically the functional significance of decreased / or change in subunit composition of the GABA_A_ receptors by determining the threshold for picrotoxin-induced seizures. Picrotoxin, an antagonist of GABA_A_ receptors that blocks the channels while in the open state, binds within the pore of the channel between the β2 and β3 subunits. These are the same subunits to which GABA and presumably taurine binds.

**Methods:**

Two-month-old male FVB/NJ mice were subcutaneously injected with picrotoxin (5 mg kg^-1^) and observed for a) latency until seizures began, b) duration of seizures, and c) frequency of seizures. For taurine treatment, mice were either fed taurine in drinking water (0.05%) or injected (43 mg/kg) 15 min prior to picrotoxin injection.

**Results:**

We found that taurine-fed mice are resistant to picrotoxin-induced seizures when compared to age-matched controls, as measured by increased latency to seizure, decreased occurrence of seizures and reduced mortality rate. In the picrotoxin-treated animals, latency and duration were significantly shorter than in taurine-treated animas. Injection of taurine 15 min before picrotoxin significantly delayed seizure onset, as did chronic administration of taurine in the diet. Further, taurine treatment significantly increased survival rates compared to the picrotoxin-treated mice.

**Conclusions:**

We suggest that the elevated threshold for picrotoxin-induced seizures in taurine-fed mice is due to the reduced binding sites available for picrotoxin binding due to the reduced expression of the beta subunits of the GABA_A_ receptor. The delayed effects of picrotoxin after acute taurine injection may indicate that the two molecules are competing for the same binding site on the GABA_A_ receptor. Thus, taurine-fed mice have a functional alteration in the GABAergic system. These include: increased GAD expression, increased GABA levels, and changes in subunit composition of the GABA_A_ receptors. Such a finding is relevant in conditions where agonists of GABA_A_ receptors, such as anesthetics, are administered.

## Background

Maintenance of the level of excitability of neurons in the central nervous system is essential to maintain homeostasis. This balance is achieved through the regulation of excitatory and inhibitory neurotransmitters. Any change in this balance can lead to hyperexcitable cells and subsequently to seizures. Possible mechanisms that may contribute to hyperexcitability include changes in ion homeostasis, ion pumps, hormones, and changes in levels/efficiency of neurotransmitters. Of these neurotransmitters, the regulation of neuron excitability by γ-aminobutyric acid (GABA), the predominant inhibitory neurotransmitter, is especially required to prevent hyperexcitability, and thus prevent seizures. Epileptogenicity is characterized by chronic hypersensitivity to sensory stimuli and thus is dependent upon the amount of hyperexcitability expressed by neurons. In a homeostatic brain, the GABAergic system plays an integral role in lowering the threshold required for an excitatory stimulus of neurons. GABA, released from presynaptic neurons, binds to the ionotropic GABA_A_ receptor, allowing chloride influx and resulting in the hyperpolarization of the postsynaptic neuron. Any perturbation of the GABAergic system, therefore, could contribute to excitability of the neuron and seizure induction.

Synthesis of GABA by glutamic acid decarboxylase (GAD) is critical for maintenance of GABA-mediated inhibition and regulating levels of excitability [1,2]. GAD exists in two isoforms, GAD65 and GAD67, both encoded by different genes [3]. Both enzymes require the coenzyme pyridoxal phosphate, with GAD65 having a more significant requirement [2,4] for regulation of activity. GAD65 appears to be an apoenzyme (lacking the coenzyme), but once the coenzyme is present, exhibits a significantly higher enzymatic activity than GAD67 [5]. GAD67 exists mainly as a holoenzyme in the cytoplasm [5]; regulation of this enzyme appears to be more associated with gene-level expression [2]. There is also abundant evidence that GAD65 expression can also be affected at the gene-level [3,6,7].

As GAD is the rate-limiting enzyme for GABA synthesis, perturbation of GAD activity would lead to GABA depletion and, subsequently, to an increase in seizure susceptibility. Isoniazid, a widely used drug to combat tuberculosis, is also and effective GAD inhibitor, leading to the rapid depletion of GABA [8-10]. Large doses of isoniazid cause severe fatal seizures in experimental animals [11]. We have previously reported that the threshold dose for induction of seizures in mice is 200 mg kg^-1^[12], and that doses higher than 200 mg kg^-1^ induce seizures of short duration and latency. Isoniazid is not GAD-specific, but also inhibits other enzymes required pyridoxal phosphate as a coenzyme. When mice are administered pyridoxal phosphate 15 min prior to treatment with isoniazid, we found that the threshold shifted to 250 mg kg^-1^ and that doses as high as 350 mg kg^-1^ delayed seizure onset and severity [12]. The data suggest that isoniazid likely competes for the pyridoxal phosphate-binding site on GAD.

Seizures can be induced by the administration of kianic acid (KA), a glutamate analogue. Treatment with KA can manifest in the GABAergic system through loss of a subpopulation of GAD-positive neurons, leading to limbic seizures [13]. Limbic seizures mostly affect the hippocampus, dentate gyrus, and entorhinal cortex [14,15]. Previously, we have reported that the threshold dose for KA is 10 mg kg^-1^[12], with doses at or above 30 mg kg^-1^ inducing fatal seizures. Taken together, both isoniazid and KA appear to negatively regulate the GABAergic system, either directly through hyperexcitability or indirectly through depletion of GABA), resulting in seizures.

We are interested, therefore, in mechanisms by which we may positively influence the GABAergic system to form a compensatory mechanism by which seizure onset and severity may be reduced. To this end, we have found that taurine may be beneficial and may work through the GABAergic system via the GABA_A_ receptor. We have previously reported that chronic supplementation of taurine in drinking water to mice increases brain excitability mainly through alterations in the inhibitory GABAergic system [12-15]. Taurine, 2-aminoethanesulfonic acid, concentrations are high in the CNS [16], especially in the neonate [17-19], but drop during development. Others and our laboratories have demonstrated a relationship between taurine and the GABAergic system. For example, there are brain region-specific levels of GAD and that GAD expression (both isoforms) is elevated in mice chronically fed taurine [12,18]. Taurine is an agonist of the GABA_A_ receptor [20,21] and activates chloride influx into postsynaptic neurons via this receptor [19]. Chronic administration of taurine to mice leads to a reduction in the β2/β3 GABA_A_ subunits [19]. Using a sub-threshold dose of isoniazid coupled with sub-threshold dose of KA, we have demonstrated that mice undergo seizures with a short latency and duration, and this combination was lethal in a majority of animals [12]. In mice chronically administered taurine prior to isoniazid/KA treatment, we demonstrated that taurine was effective in reducing the severity of seizures as latency was significantly increased and mortality significantly decreased [12].

Together, our data suggest that taurine interacts directly with the GABAergic system, likely via the GABA_A_ receptor. To further test this hypothesis, here we used a potent GABA_A_ antagonist, picrotoxin. Picrotoxin binds to the β2/β3 subunits of the GABA_A_ receptor, the same subunits demonstrated to be reduced by chronic exposure to taurine. Here we describe the efficacy of taurine in decreasing picrotoxin-induced seizures.

## Methods

### Pharmacological agents

Picrotoxin was dissolved in isotonic saline at 3 mg/ml. All mice used in this study were two-month-old FVB/NJ males and all injections were subcutaneous. For taurine-fed mice, taurine was dissolved in water at 0.05%, and this solution was made available to the mice in place of drinking water for 4 weeks beginning at 4 weeks of age. For taurine-injected mice, mice were administered 43 mg kg^-1^ subcutaneous 15 min prior to picrotoxin treatment. All mice were housed in groups of three in a pathogen-free room maintained on a 12 hr light/dark cycle and given food and water ad libitum. All procedures were approved by the Institutional Animal Care and Use Committee of the College of Staten Island/CUNY and were in conformity with National Institutes of Health Guidelines.

### Behavioral analysis

Animals were put into individual cages the day before the experiments. After treatment, animals were transferred to clear animal cages and videotaped for 4 h. Seizures were scored by two independent observers who were unaware of the treatment. The observers were asked to look for the following stereotypical behaviors: motionless stare, rearing and falling, clonic convulsions, tonic-clonic seizures (status epilepticus) and death. The occurrence of these behaviors, the time from injection to initiation of the behavior (latency) and the duration of the convulsions are measures of seizure severity. Saline-injected animals did not show any seizure behavior.

## Results

### Behavioral analysis

**Figure 1:**
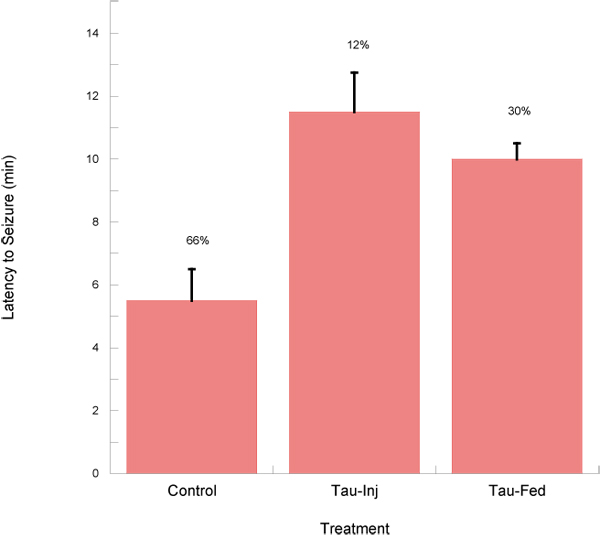
**Latency to seizures** Two-month-old male mice treated with 5 mg kg^-1^ picrotoxin presented with short latency periods (control) that were also of short duration. Seizures were nearly always fatal (66%). Treatment with taurine significantly increased latency and duration, whether route of administration was injection 15 min prior to picrotoxin injection (Tau-Inj) or chronic feeding of taurine (Tau-Fed). In both cases, taurine significantly improved survivability (P<0.05).

Following picrotoxin injection, control mice exhibited a short latency period to the onset of seizures (Figure 1). The duration of these seizures were short, and in two thirds of the control mice, seizures were fatal. In the taurine-injected mice, the latency was significantly longer (P<0.001) as were the durations (Figure 1). Further, mortality rate in these mice were also significantly less (12% p<0.001), suggesting that taurine was protective of the effects of picrotoxin via the GABA_A_ channel. Similarly, chronic administration of taurine also significantly reduced the effects of picrotoxin, as the latency and duration of seizures were also longer (P<0.05) (Figure 1). Chronic administration also significantly improved survivability compared to controls. The data suggests that taurine may act either at the picrotoxin-binding site or at the GABA binding site of the GABA_A_ receptor. Alternatively, taurine could mediate it protective effects against picrotoxin-induced seizures through activation of taurine receptor [22].

## Discussion

Picrotoxin is a potent antagonist of the GABA_A_ receptor. Binding of picrotoxin to β2/β3 subunits of the receptor effectively blocks the chloride channel, resulting in a post-synaptic neuron that is more easily excitable and prone to hyperexcitability. As such, picrotoxin-induced toxicity is epileptogenic [10,23-25]. There is compelling evidence that taurine interacts with the GABAergic system via the GABA_A_ receptor [19,25-30]. Taurine as also been shown to activate a taurine receptor [22], but the molecular identity of this receptor has not been fully characterized. Chronic taurine administration results in improved chloride conductance while selective depression of β2/β3 subunits expression occurs [19], the same subunits to which picrotoxin binds [31]. Taurine therefore maintains the integrity of the chloride channel via binding to the receptor. The site to which taurine binds, however, remains elusive. The data here suggests that taurine may bind to the GABA binding site of the receptor, keeping the channel open. In both taurine-fed and injected mice, hyperexcitability was diminished, as demonstrated by the longer latency and duration of seizures. If taurine binds to the GABA binding site, the receptor would remain open as long as taurine was present. This scenario could explain the acute taurine administration data: taurine binds to the GABA_A_ receptor and allows the cells to become hyperpolarized and thus resistant to picrotoxin-induced seizures. For the chronically fed taurine animals, the taurine would most likely be sequestered by neurons, forming intracellular pools of taurine that would primarily be used for osmoregulation of the neurons [32-36]. In the taurine-fed mice, the administration of picrotoxin could signal a release of intracellular stores of taurine, which could bind to the GABA binding site and open the channels. An alternative explanation of these findings would be the activation of the taurine receptor [22] or a synergistic effect between the GABA_A_ and the taurine receptor could explain the selective resistance to mice to picrotoxin-induced seizures.

## Conclusions

Taurine administration may interact with the GABAergic system at two points. First, taurine may interact at the level of the enzyme GAD. Chronic administration of taurine to mice leads to an increase in GAD levels (both isoforms) in GABAergic neurons. This in turn leads to an increased expression in GABA in presynaptic neurons. Second, taurine interacts at the level of the GABA_A_ receptor. Binding of taurine to the receptor increases chloride influx into the cell, hyperpolarizing the postsynaptic neuron to reduce excitability. Chronic administration of taurine also influences the expression of the β2/β3 subunits of the GABA_A_ receptor, which in turn may influence the expression of GAD in the presynaptic neuron via a feedback mechanism. The data from this and previous studies provide strong evidence for the neuroprotective role of taurine in the GABAergic system.

## Competing interests

The authors have no competing interests.

## Authors' contributions

WJL participated in the design of the study, and drafted the manuscript. AEI conceived of the study, performed the statistical analysis and participated in its design and coordination as well as edited the manuscript. Alexandra Marsillo video recorded seizures and helped in recording data of seizures. All authors read and approved the final manuscript.

## References

[B1] Martin DL Rimvall K Regulation of gamma-aminobutyric acid synthesis in the brain. J Neurochem 1993 60 395 407 10.1111/j.1471-4159.1993.tb03165.x 8419527

[B2] Soghomonian JJ Martin DL Two isoforms of glutamate decarboxylase: why? Trends Pharmacol Sci 1998 19 500 505 10.1016/S0165-6147(98)01270-X 9871412

[B3] Soghomonian JJ Laprade N Glutamate decarboxylase (GAD67 and GAD65) gene expression is increased in a subpopulation of neurons in the putamen of Parkinsonian monkeys. Synapse 1997 27 122 132 10.1002/(SICI)1098-2396(199710)27:2<122::AID-SYN3>3.0.CO;2-G 9266773

[B4] Greif KF Tillakaratne NJ Erlander MG Feldblum S Tobin AJ Transient increase in expression of a glutamate decarboxylase (GAD) mRNA during the postnatal development of the rat striatum. Dev Biol 1992 153 158 164 10.1016/0012-1606(92)90100-U 1516745

[B5] Battaglioli G Liu H Martin DL Kinetic differences between the isoforms of glutamate decarboxylase: implications for the regulation of GABA synthesis. Journal of Neurochemistry 2003 86 879 887 10.1046/j.1471-4159.2003.01910.x 12887686

[B6] Bowers G Cullinan WE Herman JP Region-specific regulation of glutamic acid decarboxylase (GAD) mRNA expression in central stress circuits. J Neurosci 1998 18 5938 5947 9671680 10.1523/JNEUROSCI.18-15-05938.1998 PMC6793042

[B7] Esclapez M Houser CR Up-regulation of GAD65 and GAD67 in remaining hippocampal GABA neurons in a model of temporal lobe epilepsy. J Comp Neurol 1999 412 488 505 10.1002/(SICI)1096-9861(19990927)412:3<488::AID-CNE8>3.0.CO;2-6 10441235

[B8] Casey RE Wood JD Isonicotinic acid hydrazide-induced changes in the metabolism of gamma-aminobutyric acid in the brain of four species. Comp Biochem Physiol B 1973 45 741 748 10.1016/0305-0491(73)90135-1 4737979

[B9] Eli M Cattabeni F Endogenous gamma-hydroxybutyrate in rat brain areas: postmortem changes and effects of drugs interfering with gamma-aminobutyric acid metabolism. J Neurochem 1983 41 524 530 10.1111/j.1471-4159.1983.tb04770.x 6875551

[B10] Vergnes M Boehrer A Reibel S Simler S Marescaux C Selective susceptibility to inhibitors of GABA synthesis and antagonists of GABA(A) receptor in rats with genetic absence epilepsy. Exp Neurol 2000 161 714 723 10.1006/exnr.1999.7302 10686090

[B11] Ruffmann C Bogliun G Beghi E Epileptogenic drugs: a systematic review. Expert Rev Neurother 2006 6 575 589 10.1586/14737175.6.4.575 16623656

[B12] El Idrissi A L'Amoreaux WJ Selective resistance of taurine-fed mice to isoniazide-potentiated seizures: in vivo functional test for the activity of glutamic acid decarboxylase. Neuroscience 2008 156 693 699 10.1016/j.neuroscience.2008.07.055 18727952

[B13] Sperk G Lassmann H Baran H Seitelberger F Hornykiewicz O Kainic acid-induced seizures: dose-relationship of behavioural, neurochemical and histopathological changes. Brain Res 1985 338 289 295 10.1016/0006-8993(85)90159-3 4027598

[B14] Ben-Ari Y Limbic seizure and brain damage produced by kainic acid: mechanisms and relevance to human temporal lobe epilepsy. Neuroscience 1985 14 375 403 10.1016/0306-4522(85)90299-4 2859548

[B15] Bruton CJ 'Status epilepticus. I: Pathogenesis'. Dev Med Child Neurol 1993 35 277 8462764

[B16] Huxtable RJ Taurine in the central nervous system and the mammalian actions of taurine. Prog Neurobiol 1989 32 471 533 10.1016/0301-0082(89)90019-1 2664881

[B17] Kuriyama K Hashimoto T Interrelationship between taurine and GABA. Adv Exp Med Biol 1998 442 329 337 9635048 10.1007/978-1-4899-0117-0_41

[B18] Sturman JA Taurine in development. Physiol Rev 1993 73 119 147 8419963 10.1152/physrev.1993.73.1.119

[B19] El Idrissi A Trenkner E Taurine as a modulator of excitatory and inhibitory neurotransmission. Neurochem Res 2004 29 189 197 10.1023/B:NERE.0000010448.17740.6e 14992278

[B20] Quinn MR Harris CL Taurine allosterically inhibits binding of [35S]-t-butylbicyclophosphorothionate (TBPS) to rat brain synaptic membranes. Neuropharmacology 1995 34 1607 1613 10.1016/0028-3908(95)00118-2 8788958

[B21] Frosini M Sesti C Dragoni S Valoti M Palmi M Dixon HB Machetti F Sgaragli G Interactions of taurine and structurally related analogues with the GABAergic system and taurine binding sites of rabbit brain. Br J Pharmacol 2003 138 1163 1171 10.1038/sj.bjp.0705134 12684273 PMC1573748

[B22] Wu JY Tang XW Tsai WH Taurine receptor: kinetic analysis and pharmacological studies. Adv Exp Med Biol 1992 315 263 268 1324594 10.1007/978-1-4615-3436-5_31

[B23] Burt GS Strain differences in picrotoxin seizure threshold. Nature 1962 193 301 302 10.1038/193301a0 13875057

[B24] DeFeudis FV Elliott KA Convulsions and the gamma-aminobutyric acid content of rat brain. Can J Physiol Pharmacol 1968 46 803 804 5681539 10.1139/y68-123

[B25] Saito S Tokunaga Y Some correlations between picrotoxin-induced seizures and gamma-aminobutyric acid in animal brain. J Pharmacol Exp Ther 1967 157 546 554 6048015

[B26] El Idrissi A Taurine improves learning and retention in aged mice. Neurosci Lett 2008 436 19 22 10.1016/j.neulet.2008.02.070 18375059

[B27] El Idrissi A Boukarrou L Splavnyk K Zavyalova E Meehan EF L'Amoreaux W Functional implication of taurine in aging. Adv Exp Med Biol 2009 643 199 206 full_text 19239150 10.1007/978-0-387-75681-3_20

[B28] El Idrissi A Messing J Scalia J Trenkner E Prevention of epileptic seizures by taurine. Adv Exp Med Biol 2003 526 515 525 12908638 10.1007/978-1-4615-0077-3_62

[B29] Chan-Palay V Ito M Tongroach P Sakurai M Palay S Inhibitory effects of motilin, somatostatin, [Leu]enkephalin, [Met]enkephalin, and taurine on neurons of the lateral vestibular nucleus: interactions with gamma-aminobutyric acid. Proc Natl Acad Sci U S A 1982 79 3355 3359 10.1073/pnas.79.10.3355 6124970 PMC346414

[B30] Louzada PR Lima AC Mendonca-Silva DL Noel F De Mello FG Ferreira ST Taurine prevents the neurotoxicity of beta-amyloid and glutamate receptor agonists: activation of GABA receptors and possible implications for Alzheimer's disease and other neurological disorders. Faseb J 2004 18 511 518 10.1096/fj.03-0739com 15003996

[B31] Chen L Durkin KA Casida JE Structural model for gamma-aminobutyric acid receptor noncompetitive antagonist binding: widely diverse structures fit the same site. Proc Natl Acad Sci U S A 2006 103 5185 5190 10.1073/pnas.0600370103 16537435 PMC1458815

[B32] Hussy N Deleuze C Desarmenien MG Moos FC Osmotic regulation of neuronal activity: a new role for taurine and glial cells in a hypothalamic neuroendocrine structure. Prog Neurobiol 2000 62 113 134 10.1016/S0301-0082(99)00071-4 10828380

[B33] Olson JE Li GZ Osmotic sensitivity of taurine release from hippocampal neuronal and glial cells. Adv Exp Med Biol 2000 483 213 218 full_text 11787600 10.1007/0-306-46838-7_23

[B34] Schaffer S Takahashi K Azuma J Role of osmoregulation in the actions of taurine. Amino Acids 2000 19 527 546 10.1007/s007260070004 11140357

[B35] Olson JE Evers JA Banks M Brain osmolyte content and blood-brain barrier water permeability surface area product in osmotic edema. Acta Neurochir Suppl (Wien) 1994 60 571 573 7976653 10.1007/978-3-7091-9334-1_158

[B36] Wade JV Olson JP Samson FE Nelson SR Pazdernik TL A possible role for taurine in osmoregulation within the brain. J Neurochem 1988 51 740 745 10.1111/j.1471-4159.1988.tb01807.x 3411323

